# Allosteric regulation of deubiquitylase activity through ubiquitination

**DOI:** 10.3389/fmolb.2015.00002

**Published:** 2015-02-05

**Authors:** Serena Faggiano, Rajesh P. Menon, Geoff P. Kelly, Sokol V. Todi, K. Matthew Scaglione, Petr V. Konarev, Dmitri I. Svergun, Henry L. Paulson, Annalisa Pastore

**Affiliations:** ^1^National Institute for Medical Research, Medical Research CouncilLondon, UK; ^2^Medical Research Council Biomedical NMR Centre, National Institute for Medical Research, Medical Research CouncilLondon, UK; ^3^Department of Pharmacology and Department of Neurology, Wayne State University School of MedicineDetroit, MI, USA; ^4^Department of Biochemistry and The Neuroscience Research Center, Medical College of WisconsinMilwaukee, WI, USA; ^5^European Molecular Biology LaboratoryHamburg, Germany; ^6^Laboratory of Reflectometry and Small-Angle Scattering, Institute of Crystallography Russian Academy of SciencesMoscow, Russia; ^7^Department of Neurology, University of Michigan Medical SchoolAnn Arbor, MI, USA; ^8^Department of Clinical Neuroscience, King's College LondonLondon, UK

**Keywords:** ataxin-3, SCA3, polyglutamine disease, deubiquitinating enzyme, cysteine protease, ubiquitin, structure

## Abstract

Ataxin-3, the protein responsible for spinocerebellar ataxia type-3, is a cysteine protease that specifically cleaves poly-ubiquitin chains and participates in the ubiquitin proteasome pathway. The enzymatic activity resides in the N-terminal Josephin domain. An unusual feature of ataxin-3 is its low enzymatic activity especially for mono-ubiquitinated substrates and short ubiquitin chains. However, specific ubiquitination at lysine 117 in the Josephin domain activates ataxin-3 through an unknown mechanism. Here, we investigate the effects of K117 ubiquitination on the structure and enzymatic activity of the protein. We show that covalently linked ubiquitin rests on the Josephin domain, forming a compact globular moiety and occupying a ubiquitin binding site previously thought to be essential for substrate recognition. In doing so, ubiquitination enhances enzymatic activity by locking the enzyme in an activated state. Our results indicate that ubiquitin functions both as a substrate and as an allosteric regulatory factor. We provide a novel example in which a conformational switch controls the activity of an enzyme that mediates deubiquitination.

## Introduction

Protein ubiquitination is a reversible post-translational modification that regulates several crucial intracellular events ranging from signaling pathways to cell cycle regulation and DNA repair (Hershko and Ciechanover, 1998). It involves the covalent attachment of the C-terminal glycine of ubiquitin to a lysine of the target protein through an isopeptide bond. Proteins can be mono-, multi- or poly-ubiquitinated. Ubiquitin itself has seven lysines plus the N-terminal amino group, which can be involved in the formation of isopeptide bonds to form poly-ubiquitin chains. Among the different chain types, K48-linked and K63-linked chains are the most studied and are linked respectively to protein degradation and to endocytotic trafficking, translation and inflammatory events (Thrower et al., 2000; Chen and Sun, 2009). Ubiquitin signaling is regulated by numerous ubiquitin ligases (E3) and deubiquitinating enzymes (DUB) that have quite different properties and substrate specificities (Ardley and Robinson, 2005; Komander et al., 2009).

A DUB that has attracted particular interest in recent years is ataxin-3, an enzyme with interesting yet still elusive properties. Ataxin-3 is responsible for the neurodegenerative disorder Spinocerebellar ataxia type 3 (SCA3), also known as Machado-Joseph disease (Paulson, 2012), which is the most common dominantly inherited ataxia and a member of the polyglutamine disease family (Kawaguchi et al., 1994; Matos et al., 2011). Ataxin-3 contains an N-terminal globular domain named Josephin, in which the enzymatic catalytic triad resides, and an intrinsically unfolded C-terminus that contains the polyglutamine tract and two or three ubiquitin interacting motifs (UIMs) depending on the isoform (Scheme 1 in Supplementary Material) (Masino et al., 2003). Josephin is a cysteine protease which preferentially cleaves ubiquitin chains with five or more repeats (Albrecht et al., 2004; Chai et al., 2004; Costa et al., 2004; Rodrigues et al., 2007). The full-length protein has preference for K63 linkages (Burnett et al., 2003; Chow et al., 2004; Weeks et al., 2011) or for K48/K63-linked poly-ubiquitin chains (Winborn et al., 2008; Todi et al., 2009), whereas the isolated Josephin domain cleaves more efficiently K48 chains (Todi et al., 2009).

The Josephin domain of ataxin-3 is unusual in several respects. Despite the typical cysteine protease fold, it contains an unusually long helical hairpin not observed in other cysteine proteases (such as papain) or in other DUBs (for example YUH1 or UCH-L3) (Nicastro et al., 2005) (Scheme 1 in Supplementary Material). It also contains at least two binding sites for ubiquitin in addition to the multiple UIMs in the C-terminus of ataxin-3. Ubiquitin binding site 1 is essential for enzymatic activity (Nicastro et al., 2010) while site 2 confers K48 ubiquitin-chain linkage preference to Josephin and overlaps with the interaction site for the ubiquitin-like domain of the HHR23 proteins (Nicastro et al., 2005, 2010). The interaction of site 2 with HHR23A/B was recently shown to regulate the cellular turnover of this protein (Blount et al., 2014). Previous measurements strongly suggest that a flexible helical hairpin separating the two binding sites plays a role in ubiquitin recognition and possibly determines substrate and ubiquitin linkage specificity (Nicastro et al., 2005, 2010).

An important open question concerns ataxin-3 enzymatic properties. Except to longer poly-ubiquitin chains, the proteolytic activity of ataxin-3 is markedly lower than most other DUB enzymes. It has been shown, however, that mono-ubiquitination has some effect in activating the enzyme (Todi et al., 2009). Lysine 117 of the Josephin domain is the preferential ubiquitination site, with mono-ubiquitination at this site being sufficient to enhance the DUB activity (Todi et al., 2010). Several E3 ubiquitin ligases (Durcan and Fon, 2013), including the C-terminus of 70kDa heat-shock protein (Hsp70)-interacting protein (CHIP), parkin, and E4B, promote ataxin-3 ubiquitination (Matsumoto et al., 2004; Jana et al., 2005; Miller et al., 2005). Lysine 117 ubiquitination is also able to suppress expanded polyglutamine-dependent degeneration in *Drosophila melanogaster* (Tsou et al., 2013), suggesting that lysine 117 ubiquitination is critical for a putative neuroprotective role of the protein. Despite increasing interest in the role of ataxin-3 ubiquitination and in how ubiquitination may influence DUB activity, little is known about the mechanism by which this modification leads to enzyme activation. Knowing this mechanism would increase our understanding of the ubiquitin pathways and provide new information on polyglutamine expansion diseases that may be translated into specific treatments.

Here, we used complementary biophysical techniques to study the enzymatic activity of Josephin and the factors influencing it. We also addressed the role of ubiquitination and its consequences at the structural level. We exploited for these studies our recent protocol that allows us to produce highly pure recombinant Josephin ubiquitinated at K117 in quantities suitable for structural studies (Faggiano et al., 2013). Our data provide a completely new understanding of the role of site 1: binding of covalently linked mono-ubiquitin locks the enzyme in an active state. Our results provide a structural explanation on how ubiquitination can directly regulate the DUB activity of ataxin-3 and enable us to propose a general regulatory mechanism that can modulate the activity of other such enzymes.

## Experimental procedures

### Protein production

The N-terminal Josephin domain of ataxin-3 (amino acids 1–182) in which all lysines but K117 were mutated to arginines (JosK117-only) was produced as previously reported (Todi et al., 2010; Faggiano et al., 2013). Recombinant wild-type Josephin was obtained with the same purification method. The W87R mutant of JosK117-only was prepared by using QuickChange Site-Directed Mutagenesis kit (Stratagene). E1 from insect cells, UbcH5a (E2, Addgene plasmid 15782) and CHIP (E3) enzymes from *E. coli* expression were all purified by affinity chromatography. Recombinant wild-type human ubiquitin was purified by anion exchange followed by gel filtration. Labeled samples for nuclear magnetic resonance (NMR) experiments were obtained by expression in minimal medium containing ^15^NH_4_Cl and ^13^C-glucose as the sole nitrogen and carbon source.

### *In vitro* ubiquitination and purification of ubiquitinated josephin

*In vitro* ubiquitination was achieved by adapting and scaling up a previous protocol (Todi et al., 2009), as described in Faggiano et al. (2013). In short, we lowered the temperature from 37°C to 25°C and reduced the salt concentration (i.e., eliminating KCl) to reduce the risk of Josephin aggregation. The reaction was carried out for 18–20 h using 50 μM JosK117-only, 1 μM E1, 8 μM UbcH5a, 1 μM CHIP, 250 μM ubiquitin, 4.5 mM ATP-MgCl_2_, in buffer 50 mM Tris pH 7.5, 0.5 mM DTT. The mono-ubiquitinated protein was then purified by anion exchange using a Hi-Trap Q HP column (GE Healthcare). After a first wash step at 0.1 M NaCl, the protein was eluted with a linear salt gradient (0.1-0.2 M NaCl) in buffer 50 mM Tris pH 7.5, 2 mM DTT.

### Circular dichroism (CD)

Far-UV CD spectra were recorded on a Jasco J-715 spectropolarimeter equipped with a Peltier temperature control system. Samples were prepared in buffer 20 mM sodium phosphate pH 6.5, 2 mM DTT.

### Activity assays with Ub-AMC and di-ubiquitin

DUB activity was measured using the fluorogenic substrate 7-amino-4-methylcoumarin ubiquitin (Ub-AMC, Boston Biochem). 1 μM Ub-AMC was added to 100 nM protein samples in buffer 20 mM Tris pH 8.0, 0.1% BSA, 1 mM DTT. The concentration of isopeptidase T was 10 nM. Experiments were performed at 25°C. Ub-AMC cleavage was monitored as a function of time measuring the increased fluorescence emission at 460 nm after excitation at 380 nm. Experiments in the presence of free ubiquitin were performed in similar conditions, without adding BSA to the buffer. Cleavage of di-ubiquitin by JosK117-only and its mono-ubiquitinated form was performed at 25°C in 50 mM Tris pH 7.5, 0.5 mM DTT, using 20 μM Josephin and 20 μM K48- and K63-linked di-ubiquitin.

### NMR measurements

Josephin samples for NMR experiments were prepared in 20 mM Na phosphate, pH 6.5, 2 mM DTT at a concentration of 250–300 μM, unless otherwise specified. Measurements were carried out at 25°C on Bruker 600 and 700 MHz, and Varian INOVA 800 MHz instruments. Backbone assignment for JosK117-only was obtained by HNCACB and CBCA(CO)NH triple resonance 3D experiments. HNCA and HN(CO)CA experiments were used for ubiquitinated JosK117-only. The assignment of ^15^N labeled ubiquitin linked to K117 on Josephin was performed by comparison with the 3D ^15^N-NOESY HSQC of free ubiquitin. Chemical shift perturbation (CSP) analysis of the changes occurring upon ubiquitination was carried out applying the following formula: Δδ_avg_ = {1/2[Δδ^2^_NH_ + (0.2Δδ_N_)^2^]}^1/2^. Relaxation experiments (^15^N T_1_, T_2_ and heteronuclear NOEs) were acquired at 700 MHz. Relaxation delays of 10, 100, 200, 400, 700, 1100, 1400, and 16, 32, 48, 63, 79, 111, 152 ms were used for T_1_ and T_2_ measurements, respectively. Data analysis was performed excluding overlapping resonances. Titrations with unlabeled ubiquitin were performed recording ^15^N HSQC spectra of 100 μM Josephin samples up to 9 equivalents of ubiquitin. ^15^N ubiquitin (50 μM) was titrated with JosK117-onlyW87R and its mono-ubiquitinated form to a maximum ratio of 2 equivalents. Cross-saturation experiments were performed at 800 MHz on perdeuterated ^15^N JosK117-only and on the same protein covalently attached to unlabeled ubiquitin on K117. Data were acquired at three different saturation times (0.5, 1.0, and 1.5 s). Protein concentration was 210 μM for JosK117-only and 230 μM for ubiquitinated JosK117-only. A 3D ^15^N-edited NOESY-HSQC (mixing time 0.1 s) carried out at 600 MHz on a ^2^H,^15^N labeled mono-ubiquitinated JosK117-only sample (labeled JosK117-only covalently linked to unlabeled ubiquitin) was used to determine intermolecular distances. The intermolecular peaks were assigned using as a reference the ^15^N-edited NOESY-HSQC of mono-ubiquitinated JosK117-only, obtained using ^15^N labeled ubiquitin linked to unlabeled Josephin.

### Structure calculations

The structure of JosK117-only was initially homology modeled on the basis of the crystal structure of the Josephin component of ATXN3L in complex with ubiquitin (3O65) and energetically minimized using the module Biopolymer of Insight II (Accelrys, Inc.). The structural coordinates of the model of JosK117-only and of ubiquitin (PDB file 1UBQ) were used to build the structure of mono-ubiquitinated Josephin by the docking software HADDOCK. The covalent bond between Josephin K117 and ubiquitin G76 was simulated imposing an unambiguous restraint between the lysine amino group and the carbonyl group of the glycine. For ubiquitin, NMR CSP data were used to define the “ambiguous interactive restraints” (AIRs) according to HADDOCK, choosing as “active” all water accessible residues with Δδ > 0.1 ppm from the average. Cross-saturation data were used to define the AIRs for Josephin: we considered as “active” all water accessible residues with percentage of attenuation >10% at saturation time of 0.5 s. For both Josephin and ubiquitin, all solvent accessible surface neighbors of active residues (within a radius of 6.5 Å) were assigned as “passive” residues. Josephin residues 52–75 and ubiquitin residues 72–76 were defined as “fully flexible.” NOEs effects observed in a ^15^N-edited 3D NOESY-HSQC were used as additional restraints.

### SAXS experiments

Synchrotron radiation X-ray scattering data were collected on the EMBL P12 beam line at the PETRA III storage ring, DESY, Hamburg. Solutions of Josephin domain of ataxin-3 were measured at protein concentrations of 1.1, 2.1, and 4.3 mg/mL using PILATUS 2M pixel detector (DECTRIS, Switzerland), sample- detector distance 3.1 m, wavelength λ = 1.25 Å, covering the momentum transfer range 0.003 < *s* < 0.45 Å^−1^ [*s* = 4π sin(θ)/λ where 2θ is the scattering angle]. To check for radiation damage, twenty 50-ms exposures were compared; no radiation damage effects were observed. The data, after normalization to the intensity of the incident beam, were averaged, and the scattering of the buffer was subtracted. The difference data were extrapolated to zero solute concentration following standard procedures. All data manipulations were performed using the program package PRIMUS (Konarev et al., 2003). Multiple runs of the program DAMMIF (Nicastro et al., 2004), a fast version of DAMMIN (Franke and Svergun, 2009), were used to produce average low resolution *ab initio* shape of the complex. Rigid body modeling was performed with the program SASREF (Petoukhov and Svergun, 2005) using the X-ray structure of the C14 mono-ubiquitinated Josephin complex (PDB code 3O65) and optimizing the orientation/position of the ubiquitin molecule relative to the Josephin domain without any contact restraints.

## Results

### Covalently linked ubiquitin binds to site 1

We first addressed the question of how the covalently bound ubiquitin interacts with Josephin. For our studies we used a Josephin mutant (JosK117-only), in which all lysines but K117 (i.e., K8, 85, 125, 128, and 166) were mutated to arginines to ensure ubiquitination solely at lysine 117 and sample homogeneity (Todi et al., 2010). This mutant was used since it is not possible to generate wild-type mono-ubiquitinated Josephin in vitro because other lysine residues are also ubiquitinated, albeit at lower frequency than K117 (Todi et al., 2010). As shown elsewhere, these mutations do not affect protein fold or DUB activity (Todi et al., 2010; Faggiano et al., 2013). We capitalized on the ability to produce large quantities of mono-ubiquitinated JosK117-only using a protocol previously described (Faggiano et al., 2013). This capability allowed us to exploit the full richness of specific labeling schemes and use NMR to obtain structural information about the position adopted by the covalently linked ubiquitin with respect to Josephin. We produced samples in which ^15^N-labeled JosK117-only was covalently linked to unlabeled ubiquitin or ^15^N-labeled ubiquitin was linked to unlabeled JosK117-only. This allowed individual analysis of the changes occurring on each of the two proteins upon formation of the isopeptide bond.

**Figure 1:**
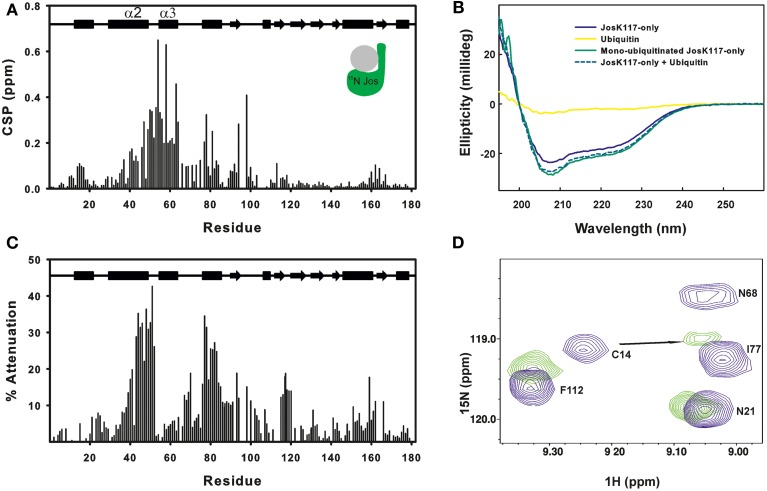
**Mapping ubiquitination on the Josephin surface**. **(A)** Chemical shift perturbation of the changes occurring on ^15^N labeled Josephin domain after ubiquitination of JosK117-only. **(B)** Far-UV CD spectra of JosK117-only (blue), mono-ubiquitinated JosK117-only (green), ubiquitin (yellow). The arithmetical sum of the spectra of JosK117-only and of free ubiquitin is reported in dashed cyan. **(C)** Cross-saturation experiment on perdeuterated ^15^N JosK117-only covalently attached to unlabeled ubiquitin on K117. **(D)** ^15^N HSQC spectra for the C14 peak before (blue) and after (green) ubiquitination.

As previously reported, ubiquitination leads to significant variations of the ^15^N HSQC spectrum of JosK117-only (Faggiano et al., 2013). Nevertheless, 65% of the amide peaks have CSP values lower than 0.05 ppm, indicating that overall the tertiary structure of Josephin is conserved after ubiquitination (Figure 1A). This conclusion was independently confirmed by far-UV CD: the spectrum of mono-ubiquitinated JosK117-only superimposes to the arithmetical sum of the spectra of JosK117-only and of free ubiquitin (Figure 1B), indicating that the secondary structure of the protein remains the same after mono-ubiquitination.

The majority of residues with larger CSP values reside between residues 40–80 and map on/near the α2/α3 helical hairpin. Cross-saturation data confirmed this pattern (Figure 1C). The residues mostly affected include the second half of helix α2, which extends away from the globular core of Josephin, the loop between helix α2 and α3, helix α3, and the long loop between helices α3 and α4 Large CSP values are also present for residues S76, I77, Q78, and S81, all of which are in site 1. In addition, ubiquitination affects the amide resonance of the catalytic cysteine (C14) which shifts from 9.24 to 9.05, whereas the nitrogen resonance experiences only a minor shift (from 119.1 ppm to 119.0 ppm) (Figure 1D). In contrast, the amide resonances of the other two residues of the catalytic triad (H119 and N134) remain mostly invariant as does Q9, the residue proposed to form the oxyanion hole (Weeks et al., 2011). Interestingly, C14, like other residues close to it (i.e., E7, S12, Q16, H17, and C18), has weak peaks in the ^15^N HSQC spectra of wild-type Josephin, JosK117-only and ubiquitinated JosK117-only. This result suggests that the catalytic cysteine and residues around it are in a conformational exchange and that ubiquitination directly influences the chemical environment of residues in this region. Since CSP indicates perturbation of the chemical environment caused by an interaction but not necessarily a direct interface, we also performed cross-saturation experiments to map surfaces in direct contact (Takahashi et al., 2000). The experiment carried out on perdeuterated ^15^N JosK117-only covalently linked to unlabeled ubiquitin confirmed that the residues involved in the interface with ubiquitin are K117, residues of site 1 and residues of helix α2 in the flexible helical hairpin (Figure 1C). The second helix forming the hairpin (helix α3) does not appear to be directly involved in the interaction. In addition, residues on the flexible loop between helix α3 and helix α4 such as G67 and D70 are at the interface with ubiquitin.

**Figure 2:**
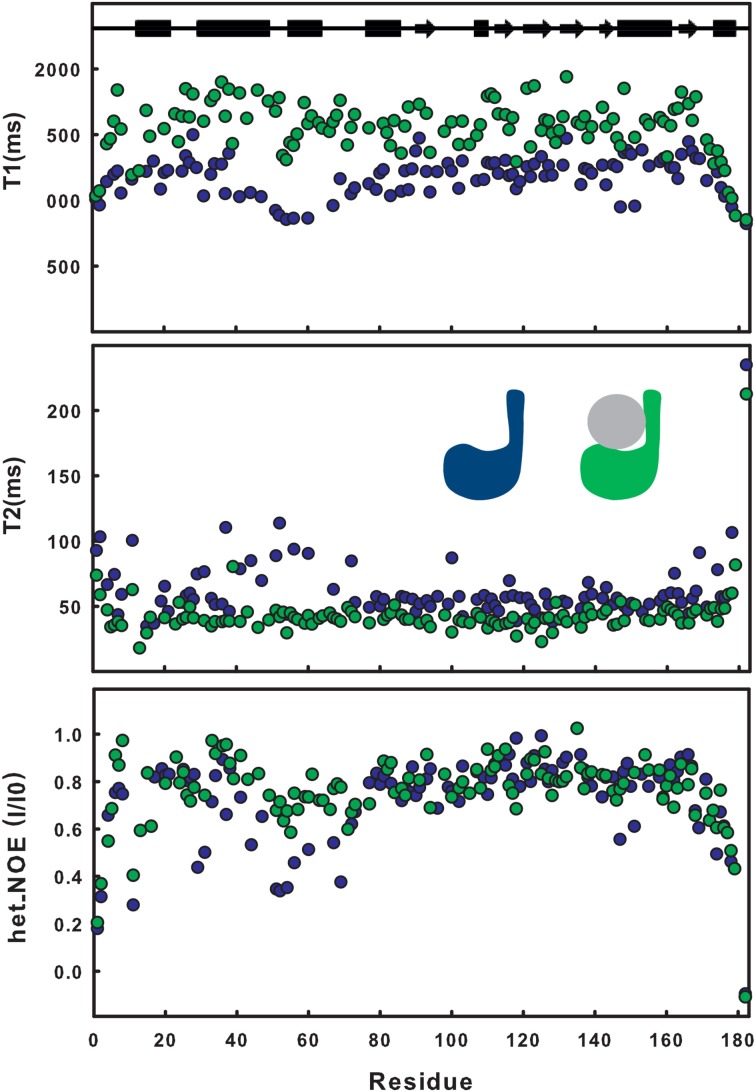
**Comparison of T_1_, T_2_, heteronuclear NOE relaxation experiments for ^15^N JosK117-only (blue) and its mono-ubiquitinated form (green)**.

The effect of ubiquitination on the dynamics of residues in or near site 1 is also evident from the NMR relaxation experiments. Ubiquitination induces a clear stiffening of the motions around the helical hairpin, as observed from the drastic change of T_1_, T_2_ and NOE values for residues of the hairpin (residues 30-75). In isolated JosK117-only, the T_1_ values and heteronuclear NOEs are significantly lower and T_2_ values higher than average (Figure 2), pointing to greater flexibility. In ubiquitinated JosK117-only, the motions of the hairpin change markedly revealing a diminished flexibility with respect to the non-ubiquitinated protein. A comparable effect was previously reported when comparing free Josephin to Josephin non-covalently bound to ubiquitin (Nicastro et al., 2009) confirming that covalently linked and free ubiquitins bind in a similar way in site 1.

Estimate of the correlation times, as calculated from the average NMR T_1_/T_2_ ratios (i.e., excluding residues 30–75), shows an increase from a value of 12.6 ns for isolated ^15^N JosK117-only (to be compared with 11.7 ns for wild-type Josephin (Nicastro et al., 2005) to a value of 16.5 ns for the mono-ubiquitinated protein. This result is in agreement with the value expected for a globular protein of the size comparable to the complex that tumbles isotropically (Maciejewski et al., 2000).

Together, these results indicate that covalently linked ubiquitin occupies site 1.

### K117-linked ubiquitin binds to Josephin through its hydrophobic patch centered on I44

**Figure 3:**
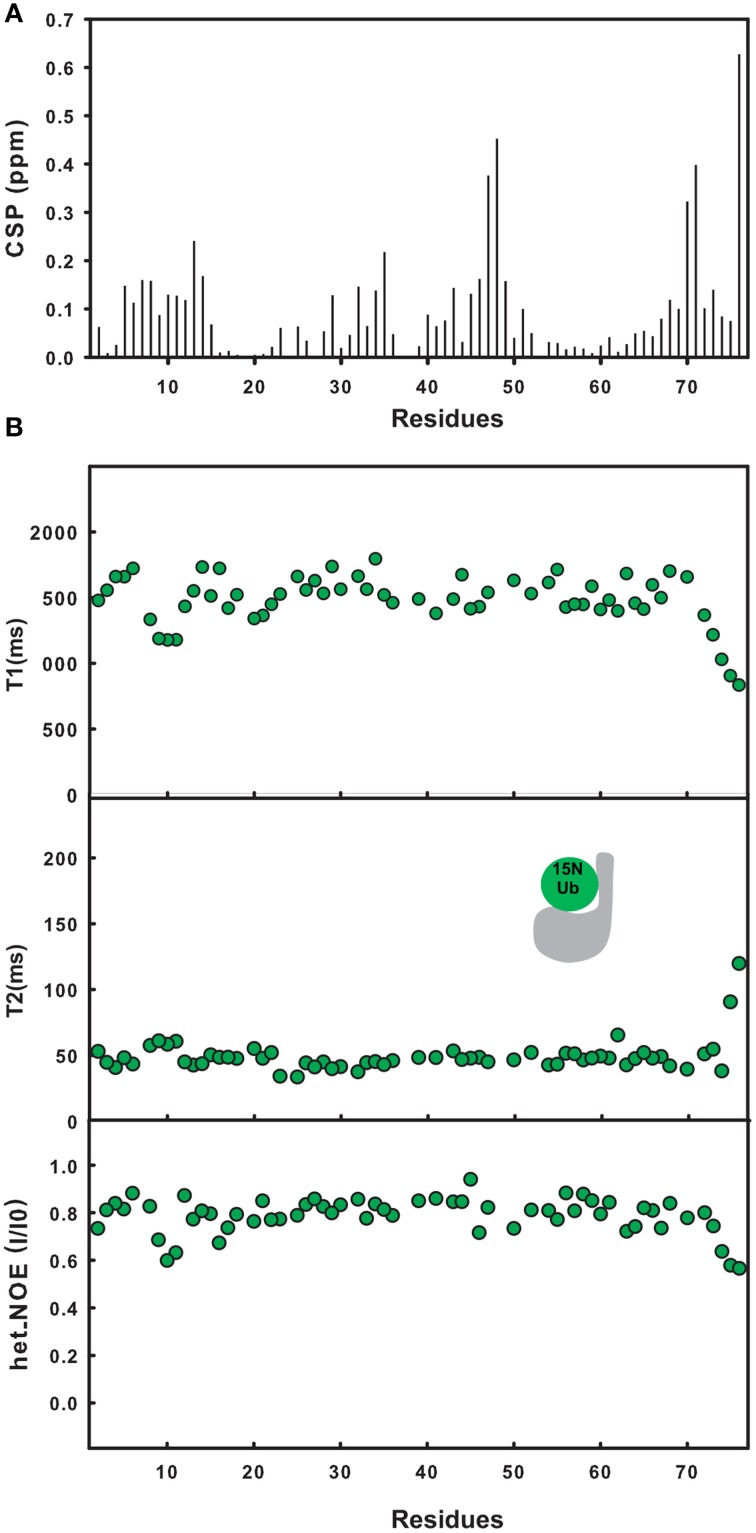
**Mapping ubiquitination on the ubiquitin surface**. **(A)** Chemical shift perturbation of the changes occurring on ^15^N ubiquitin after covalent linkage with JosK117-only. **(B)** T_1_, T_2_, heteronuclear NOE relaxation experiments for ^15^N ubiquitin covalently linked to JosK117-only (dark green).

To determine the orientation of ubiquitin in the covalent complex,^15^N ubiquitin was in turn covalently linked to unlabeled JosK117-only. As observed above for Josephin, the ^15^N HSQC spectrum of labeled ubiquitin changes significantly when it interacts with unlabeled Josephin (Faggiano et al., 2013). The residue with the largest CSP value is G76, as expected for covalent modification via this residue. Large CSP values are also observed for G47, L48, V70, and L71 (Figure 3A). Significant changes occur also for residues close in sequence to L8 and I44. The CSP profile is similar to that obtained after binding of free ^15^N ubiquitin to site 1 of Josephin in the W87K mutant which silences site 2 (Nicastro et al., 2009). The residues involved in the interface, with the exception of G76, lay on the hydrophobic surface of ubiquitin centered on I44.

To further substantiate our results, we measured the dynamical parameters of covalently-linked ubiquitin (Figure 3B). The correlation time calculated from the average T_1_/T_2_ ratio is 15.2 ns, in excellent agreement with that calculated for labeled mono-ubiquitinated JosK117-only and appreciably larger than that expected for free ubiquitin (4.1 ns, Schneider et al., 1992). These results confirm that the two molecules tumble together in solution as a compact globular assembly.

Taken together, the NMR data indicate that the covalently linked ubiquitin interacts with JosK117-only through the canonical exposed hydrophobic patch formed by L8, I44, and V70.

### Intermolecular NOEs confirm that site 1 is at the interface with ubiquitin

**Figure 4:**
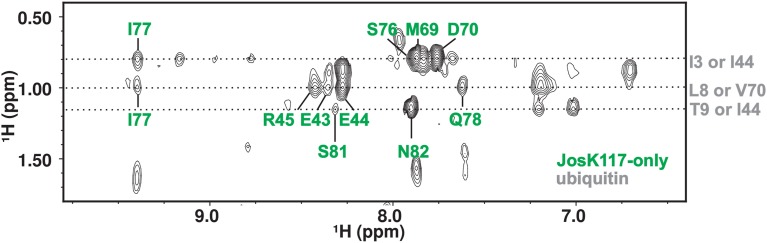
**2D projection of the ^15^N-edited NOESY-HSQC in the H-H_ind_ dimension showing the quality of the observed intermolecular NOE effects**.

To substantiate further the chemical shift perturbation and cross saturation data, we acquired a 3D ^15^N-edited NOESY-HSQC on mono-ubiquitinated JosK117-only (having the Josephin moiety ^2^H,^15^N labeled linked to unlabeled ubiquitin) to obtain intermolecular distance restraints. Perdeuteration of JosK117-only resulted crucial to obtain intermolecular NOEs. In contrast, filtered ^15^N- or ^13^C-edited NOESY-HSQC experiments on labeled JosK117-only linked to unlabeled ubiquitin were unsuccessful due to low sensitivity (they only confirmed attachment of ubiquitin to K117). The high level of deuteration of the sample was controlled by monodimensional proton NMR, ruling out the possibility of observing Josephin intramolecular NOEs in the ^15^N-edited NOESY-HSQC (data not shown). We observed a number of intermolecular NOE effects (Figure 4). The assignment of the residues of Josephin involved is unambiguous: the effects correspond to the Josephin amide protons of E43, E44, R45, M69, D70, S76, I77, Q78, S81, and N82. Assignment of the ubiquitin resonances has some ambiguity but all refer to side chain aliphatic protons of residues that, except for I3, reside in the I44 hydrophobic patch confirming that, for ubiquitin covalently linked to K117, this surface is involved in additional non-covalent binding to Josephin (Table 1).

**Table 1:** **List of AIRs, NOEs and other restraints used for the docking with HADDOCK**.

**AIRs**	**NOEs between Jos backbone amides and ubiquitin side chains**	**Jos K117-ubiquitin G76 linkage**	**Fully flexible segments**
Jos: 43, 44, 47, 48, 50, 51, 67, 70, 77, 78, 93, 117 Ub: 6, 8, 10, 11, 12, 34, 35, 46, 47, 49, 71, 76	(69, 70, 76, 77)–(3 or 44)^a^ (43, 44, 45, 77, 78)–(8 or 70) (81, 82)–(9 or 44)	distance restraint between Jos K117 N^ε^ and ubiquitin G76 carboxyl	Jos 52–75 ubiquitin 72–76
cross-saturation (Jos) CSP (ubiquitin)	^15^N edited NOESY-HSQC	Mass spectrometry, NMR	NMR relaxation experiments

*In the second row are listed the effects observed, in the third the technique by which they have been obtained. JosK117-only is abbreviated as Jos*.

a *The initial assignment ambiguities were resolved at the end of the calculations with the selection of only one possible assignment. This excluded I3 and V70*.

These results thus confirm that site 1, helix α2, and the loop between α3 and α4 are at the interface with ubiquitin.

### Modeling the structure of the complex

**Figure 5:**
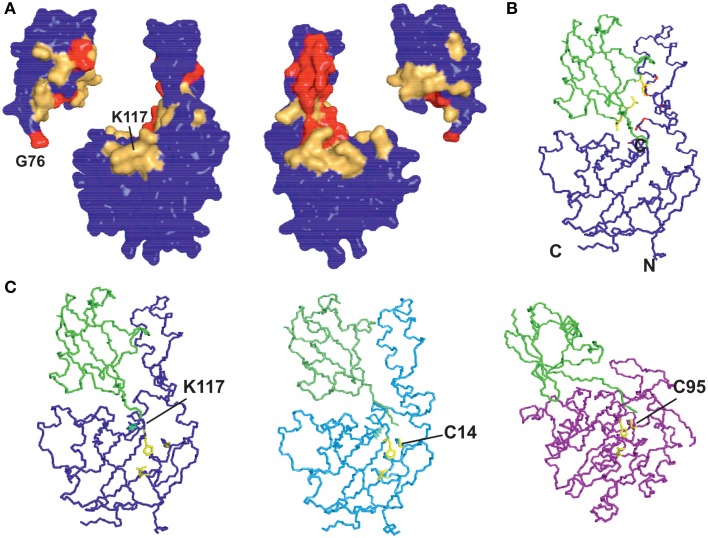
**Restraints used in the calculation and resulting structure**. **(A)** Mapping the cross saturation effects and CSP on the structures of Josephin and ubiquitin, respectively. The color coding used is the following: cross saturation on Josephin is indicated in red and orange for values of attenuation >30% and 10–30%, respectively. Values of CSP >0.3 ppm and 0.1–0.3 ppm are marked in red and orange on the surface of ubiquitin. **(B)** Mapping intermolecular NOE effects between JosK117-only and ubiquitin on the best HADDOCK model in terms of energy and restraint violations. In yellow are shown the side chains of the ubiquitin residues selected in the calculation (L8, T9, I44). In red are marked the involved (unambiguous) JosK117-only residues. **(C)** Comparison of the model of mono-ubiquitinated JosK117-only obtained using the software HADDOCK (left) with the crystal structures of the Josephin-like domain from ATXN3L (center, 3O65) and of the DUB UCH-L3 (right, 1XD3), both linked to ubiquitin by the catalytic cysteine (explicitly indicated). The structure of the UCH-L3/ubiquitin assembly is thought to mimic that of the reaction intermediate and suggests that, despite specific differences, ubiquitin binds DUB enzymes adopting approximately equivalent orientations as respect to the catalytic triads.

We modeled ubiquitinated JosK117-only on the basis of NMR CSP, cross-saturation data and intermolecular NOEs (Table 1 and Figures 5A,B). The NOE-based distances, which involve unambiguous Josephin resonances but ambiguous ubiquitin resonances, were inserted as ambiguous restraints allowing the program to discriminate between the possible assignments for each ubiquitin resonance. The distances were all uniquely assigned to a specific ubiquitin group during the iterative procedure. The final solutions can be grouped in two similar clusters of structures, one of which is statistically predominant (>75% of the structures) and is significantly better in terms of overall energy, HADDOCK score and restraint violations (Table 2). The intermolecular NOE restraints (distance ranging from 2.0 to 6.0 Å) were satisfied in all structures of cluster 1. The relative orientation of the two molecules and the position of ubiquitin in the model (Figure 5C, left) are remarkably similar to that of the crystal structure of the ataxin-3 like protein ATXN3L covalently ubiquitinated on the active site C14 (Figure 5C, center) (Weeks et al., 2011) despite the different and independent approaches adopted to obtain the structures. The only main differences are the position of the flexible loop between helix α3 and α4 in ataxin-3 and the orientation of the ubiquitin C-terminus: the loop moves toward the covalently linked ubiquitin. In the X-ray structure of ATXN3L, the C-terminus of ubiquitin is covalently attached to C14 and, to reach this side chain, inserts in between the backbone atoms of F74 and E118. In the model of mono-ubiquitinated JosK117-only, the C-terminus is rotated by approximately 45 degrees to reach the more exposed side chain of K117 that is situated in the main sub-domain on a β-hairpin above the active site. Other differences may well be within the resolution of the HADDOCK model. Interestingly, the mode of interaction also broadly agrees with other DUB complexes with ubiquitin (Figure 5C, right).

**Table 2:** **Statistics for the analysis of the structures of the covalent complex calculated by HADDOCK**.

**Critera**	**Cluster 1**	**Cluster 2**
HADDOCK score	−133.4 ± 5.2	−86.6 ± 3.1
Cluster size	160	40
RMSD from lowest-energy structure	1.5 ± 0.9	8.8 ± 0.9
Van der Waals energy	−80.8 ± 6.2	−88.0 ± 5.5
Electrostatic energy	−468.0 ± 19.0	−270.0 ± 61.7
Desolvation energy	40.1 ± 3.8	26.7 ± 7.0
Restraints violation energy	9.3 ± 11.4	286.5 ± 106.39
Buried Surface Area	2602.8 ± 69.3	2395.7 ± 67.3
Z-score	−1.0	1.0

These results indicate that the overall modality of interaction with ubiquitin is independent from the specific anchoring mode.

It is also important to notice that, in the model of mono-ubiquitinated Josephin, the residues of the catalytic triad (C14, H119, and N134) are exposed to the solvent, hence still available for the recognition and binding of the isopeptide bond in the substrate. Therefore, the covalent binding of ubiquitin on K117 and the non-covalent coordination to site 1 do not impair the access of the substrate cleavable group to the active site.

### Covalent ubiquitination competes with free ubiquitin for binding to site 1

**Figure 6:**
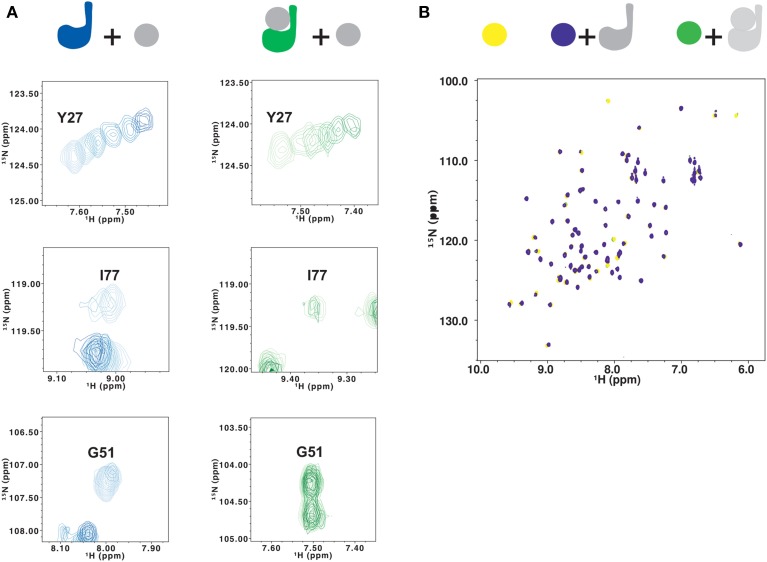
**Testing the effects of ubiquitination on lysine 117 by NMR**. **(A)** Titration of ^15^N JosK117-only (left, from light to dark blue) and of mono-ubiquitinated JosK117-only obtained using ^15^N labeled Josephin (right, from light to dark green) with free ubiquitin. Residues shown are Y27 (top), I77 (center) and G51 (bottom). **(B)** Titration of ^15^N ubiquitin (yellow) with JosK117-only W87R mutant (blue) and its mono-ubiquitinated form (green). The color scheme is indicated with a cartoon of the proteins colored to match the spectra.

To determine if the surface around site 1 is still available for substrate binding in ubiquitinated Josephin, we titrated ^15^N-labeled JosK117-only covalently linked to unlabeled ubiquitin with free ubiquitin. As a control, non-ubiquitinated ^15^N-labeled JosK117-only was titrated with free ubiquitin. In the ^15^N HSQC spectra, the resonances of residues in site 2, centered on W87, move upon addition of ubiquitin for both JosK117-only and its ubiquitinated form (Figure 6A, top). The estimated affinity constants for ubiquitin binding to site 2 are 330 and 220 μM for JosK117-only and its ubiquitinated form, respectively. The two values are comparable within experimental error, given the relatively low affinities. These results confirm that ubiquitination does not influence the availability of site 2, consistent with a previous report (Todi et al., 2010).

We observe very different results for residues in site 1 and the α2/α3 helical hairpin (i.e., I77, Q78, S81,G51, G52, T54, T60, and Q64). In JosK117-only, the resonances of these residues broaden to disappearance upon titration with ubiquitin, indicating that they are in an intermediate exchange regime. The same resonances are much less affected by titration with free ubiquitin in the spectra of mono-ubiquitinated JosK117-only (Figure 6A, center and bottom). This confirms that, in the mono-ubiquitinated protein, the helical hairpin is less readily available for binding free ubiquitin, in agreement with site 1 already being occupied.

We then titrated ^15^N ubiquitin with an unlabeled W87R mutant of JosK117-only (JosK117-onlyW87R) which markedly impairs ubiquitin binding to site 2 (Nicastro et al., 2009). This way, we could monitor only the chemical shift variations on ^15^N-labeled ubiquitin caused by binding to site 1. The CSP plot after addition of two equivalents of JosK117-onlyW87R confirmed that the interface involved in binding is the canonical I44 patch, confirming what we previously reported (Nicastro et al., 2010). The peaks corresponding to G47 and D32 disappear. Other peaks, including L8, I13, K48, V70, L71, and L73, shift. Addition of the same equivalents of unlabeled ubiquitinated JosK117-onlyW87R induced much smaller chemical shift variations (Figure 6B). This can be explained by a decreased affinity for recognition of ubiquitin in ubiquitinated Josephin.

Taken together, these data indicate that covalently linked ubiquitin competes with free ubiquitin, reducing its ability to bind to site 1.

### Validation of the mono-ubiquitinated JosK117-only model by SAXS

**Figure 7:**
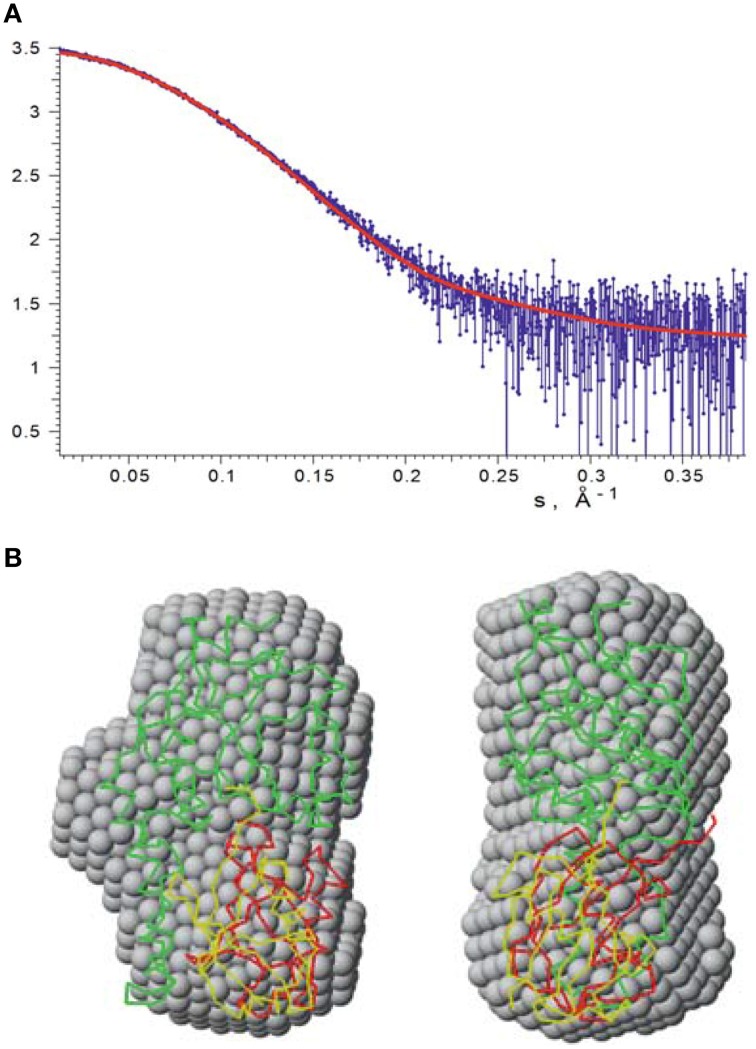
**SAXS data recorded on mono-ubiquitinated JosK117-only**. **(A)** The experimental raw data are shown in blue. Fit with our HADDOCK lowest energy model is indicated by a red line. **(B)** The lowest energy HADDOCK model of mono-ubiquitinated JosK117-only (shown as green and red carbon alpha traces) and the rigid body model (green and yellow traces) superimposed with the averaged *ab initio* bead model (gray spheres). The right view is rotated 90° counterclockwise.

We independently validated the structure of the mono-ubiquitinated JosK117-only complex by SAXS. We acquired data for isolated JosK117-only and for the complex (Figure 7A). The estimated apparent molecular mass (MM_exp_) and hydrated particle volume (*V_p_*) for JosK117-only tightly reproduced previous datasets collected for wild-type Josephin (Nicastro et al., 2006). The MM_exp_ and *V_p_* of the mono-ubiquitinated JosK117-only complex are distinctly different from those of the isolated species: the estimated molecular mass (MMexp = 26 ± 3 kDa) and hydrated particle volume (Vp = 48 ± 4 nm^3^) clearly corresponds to the 1:1 stoichiometry of the complex.

The ab initio and rigid body modeling of the mono-ubiquitinated JosK117-only data yielded consistent shapes (Figure 7B). At the same time our HADDOCK model (Figure 7B) provides the fit of similar quality as the rigid body model (Figure 7A, chi value 1.14) and overlaps with the ab initio shape. The orientation of the ubiquitin molecule relative to the Josephin domain in the rigid body model of course differs from that of the HADDOCK model. This is not surprising as no contact restraints were applied during the rigid body modeling. The fact that the positions of the ubiquitin molecule in both models are matching each other, clearly and independently endorses that the K117 covalently bound ubiquitin is positioned in site 1.

### Josephin is also activated by non-covalently bound ubiquitin

The observation that mutations at site 1 abolish enzymatic activity, together with the structural data reported here on K117 mono-ubiquitinated Josephin, suggest that ubiquitin bound to site 1 behaves as a conformational switch that brings the protein into an activated state that can only be reached when site 1 is occupied. Based on our results, the role of ubiquitin in site 1 is not that of hosting the substrate but rather of locking the protein into an active conformation. This model predicts that free ubiquitin also could have an activating effect. To validate this prediction, we probed the effect of ubiquitin on the Josephin enzymatic activity using a fluorimetric assay employing Ub-AMC, a ubiquitin derivative in which a fluorescent probe is attached to the protein C-terminus (Dang et al., 1998). The fluorescence assay allowed us to quantify for the first time the degree of activation due to mono-ubiquitination.

**Figure 8:**
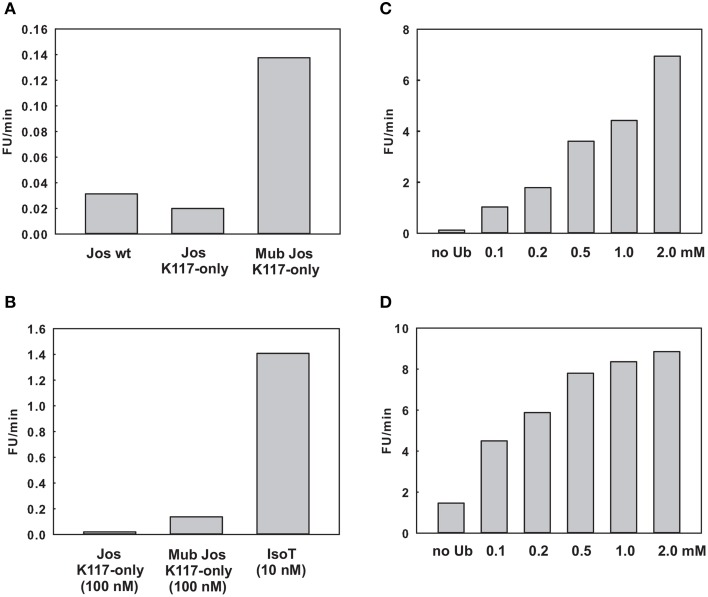
**Effect of ubiquitin on the enzymatic kinetics**. **(A)** Comparison of the cleavage rate of ubiquitin-AMC (expressed in relative fluorescence units per minute) by wild-type Josephin, JosK117-only, and mono-ubiquitinated JosK117-only. **(B)** Comparison of the cleavage rate of JosK117-only and mono-ubiquitinated JosK117-only with that of another typical DUB, isopeptidase T, at a concentration 10 times lower. **(C,D)** Enzymatic activity respectively of JosK117-only and mono-ubiquitinated JosK117-only in the presence of increasing concentrations of free ubiquitin.

**Figure 9:**
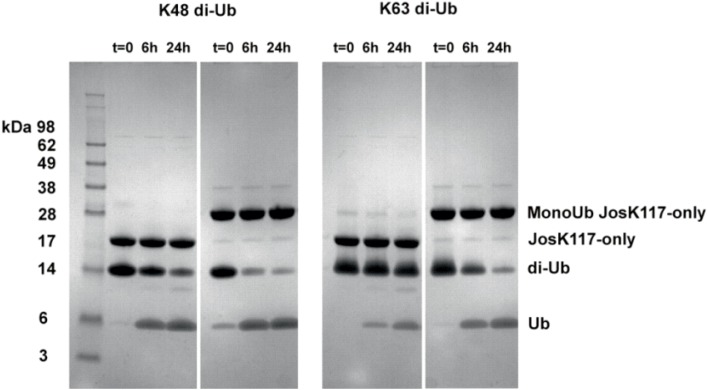
**Cleavage of K48 (left) and K63 (right) linked di-ubiquitin by JosK117-only and its mono-ubiquitinated form**. The faint bands are impurities likely from small amounts of di-ubiquitinated protein that became visible because of prolonged (overnight) staining with InstantBlue (Expedeon).

As a preliminary control, we compared the DUB activity of wild-type Josephin, JosK117-only and mono-ubiquitinated JosK117-only. The mutant JosK117-only has a behavior indistinguishable from wild-type Josephin confirming that mutations eliminating all other lysines in the protein do not affect the enzymatic activity. We detected a ~7-fold increase in the cleavage rate of Ub-AMC for mono-ubiquitinated Josephin (Figure 8A). Covalent mono-ubiquitination activates Josephin also toward cleavage of K48- and K63-linked di-ubiquitin (Figure 9). As with Ub-AMC, cleavage is anyway very ineffective: equimolar concentrations of enzyme and substrate are required to observe the activity. However, the evidence that mono-ubiquitination increases the activity not only toward longer ubiquitin chains (Todi et al., 2010) but also toward smaller substrates suggests that there is a common mechanism of activation, independent from the length of the substrate.

As a positive control to ensure that the observed low activity is not due to the conditions of our assay, we compared the Josephin activity with that of another well characterized DUB, isopeptidase T. We had to use ten-fold higher enzyme/substrate molar ratios to observe cleavage by Josephin (10 nM isopeptidase T versus 100 nM Josephin were used to cleave 1 μM Ub-AMC). Concentrations 10 times lower of isopeptidase T produce a much stronger effect, demonstrating how ineffective Josephin is on this substrate (Figure 8B). Accordingly, it was previously reported that Ub-AMC is unsuitable for rigorous Michaelis-Menten kinetic studies of Josephin as its enzymatic Km value exceeds its solubility (Weeks et al., 2011).

Finally, we probed the activity of JosK117-only at increasing concentrations of non-covalently attached ubiquitin (Figure 8C). The presence of a large excess of free ubiquitin (at concentrations ranging from 1000 to 40000 fold with respect to the substrate Ub-AMC) induced activation toward Ub-AMC cleavage. The effect becomes stronger with increasing concentrations of ubiquitin and reaches a plateau, indicating specific binding of free ubiquitin to Josephin. The addition of 2 mM ubiquitin induced a ~56-fold increase of the activity of JosK117-only. Mutation of site 2 (W87R) does not influence the effect (Figure S1 of Supplementary Materials), pointing to a crucial role of site 1 in the activation mechanism. On the contrary, mutation of site 1 (I77R/Q78R) impairs completely Ub-AMC cleavage, also in the presence of free ubiquitin (Figure S2 of Supplementary Materials). These data confirm our model and indicate that binding of ubiquitin to site 1 contributes to the activation of Josephin, supporting the idea that mono-ubiquitinated Josephin has enhanced activity due to the binding of the covalently linked ubiquitin to site 1.

Rather surprisingly, we observed a similar effect also with mono-ubiquitinated JosK117-only at increasing concentrations of non-covalently attached ubiquitin (Figure 8D). The addition of 2 mM ubiquitin caused a 6-fold increase of the rate of cleavage of Ub-AMC by mono-ubiquitinated JosK117-only. We can exclude the presence of significant contamination of the sample with unmodified protein that could explain this effect: the ion exchange purification protocol allows the separation of the two components of the ubiquitination mixture (Faggiano et al., 2013). A possible explanation is that, given the large excess of free ubiquitin added, free ubiquitin is able to displace the covalently linked ubiquitin and produce an activation effect because of the increasingly effective concentration of ubiquitin. In alternative, it is possible that Josephin could host other ubiquitin binding sites with very low affinity (millimolar range) which could be responsible for the effect of free ubiquitin on the activity of the enzyme. We plan to investigate this point further. Raw data for the kinetics are reported in Figure S3 of Supplementary Materials.

## Discussion

Understanding how DUB enzymes work and are regulated is of great interest if we wish to gain insights into the complexity of the ubiquitin code. Ataxin-3 is a particularly interesting DUB because it plays a central role in a neurodegenerative disease, interacts with multiple ubiquitin ligases, and regulates ubiquitin chain production and proteasomal degradation of proteins (Matos et al., 2011). As compared to other DUB enzymes, ataxin-3 also possesses unusual enzymatic properties including low intrinsic activity.

Here, we have studied how ubiquitination of K117 affects the Josephin domain and investigated the mechanism that leads to the previously described activation of ataxin-3 (Todi et al., 2009, 2010). Ubiquitination of Josephin is a stable modification as previously showed: ubiquitinated ataxin-3 is stable overnight and unmodified ataxin-3 is unable to deubiquitinate catalytically inactive ubiquitinated ataxin-3 (Todi et al., 2009, 2010). These data suggest that other DUBs may revert this post-translational modification. It will be interesting to determine how this regulatory cycle comes to completion.

In principle, mono-ubiquitination could lead to two possible scenarios: the covalently attached ubiquitin could be flexible or pack against Josephin. We demonstrated that ubiquitination of K117 does not influence the overall structure of the Josephin domain and that the covalently linked ubiquitin sits in site 1, adopting a structure very similar to that observed in the crystal structure of a closely related Josephin covalently attached to ubiquitin through the catalytic C14 cysteine (Weeks et al., 2011). The linkage between K117 and ubiquitin is in a position equivalent to that observed in this structure. This conclusion is strongly supported by CSP, the more direct cross-saturation and NOE data and by SAXS. We observed chemical shift perturbation very similar to that observed when adding free ubiquitin to Josephin (Nicastro et al., 2009). We could rule out intermolecular effects by measuring the correlation time which corresponds to a species of ~30 kDa, a molecular weight that is compatible with the monomeric complex. Any other scenario would appreciably increase the correlation time. Intramolecular rather than intermolecular effects between two covalently linked chains are also expected from the well accepted knowledge that linking two chains favors their direct interaction and significantly increases the chances of their encountering for entropic reasons. This concept is for instance widely used in drug design (Harner et al., 2013). In further support of our conclusions is the observation that occupancy of site 1 by the covalently attached ubiquitin results in a reduction of the hairpin flexibility similar to what was previously observed by NMR and molecular dynamics studies upon binding of non-covalently bound ubiquitin (Nicastro et al., 2009). Together, these observations cannot be explained differently than by concluding that the covalently bound ubiquitin occupies site 1 of Josephin.

In light of these new data, we propose a revised role for site 1 in ataxin-3 DUB activity: contrary to our previous belief, instead of or in addition to binding the ubiquitin substrate and “coordinating” it for attack by the catalytic triad, occupation of site 1 by mono-ubiquitin “locks” the catalytic domain into an active mode.

Ataxin-3 thus provides a novel example of enzyme regulation in which the interaction of the substrate with a substrate-binding site distinct from the catalytic site functions as a regulatory switch. This implies a feedback regulation of this enzyme in which the substrate is at the same time an allosteric regulator. Regulation of DUB activities by their binding to ubiquitin has been reported for other such enzymes (e.g., USP7) (Hu et al., 2002). However, the regulatory mechanism supported by our current findings has not been described before for a protease. Our observations fit well also with the suggestion put forward by Komander and colleagues that ubiquitination could allosterically regulate protein function (Komander, 2009; Komander et al., 2009). Another example of such a mechanism is the activation of cullin SCF ligases by NEDDylation (Duda et al., 2008). In this case, activation is caused by a conformational change that locks this E3 ubiquitinating enzyme into an active conformation. We predict that more such examples will be found, progressively revealing the complex modalities by which post-translational modifications mediate enzyme regulation.

A possible explanation of our findings and given that, when silencing site 2, mono-ubiquitinated Josephin remains enzymatically as active as the wild-type protein, is that the domain could contain a third yet unidentified ubiquitin binding site. The additional site could bind ubiquitin more weakly than site 1 and 2 since no obvious evidence of it was found in our titrations of Josephin with mono-ubiquitin (Nicastro et al., 2005, 2010). This hypothesis would explain why Ub-AMC and di-ubiquitin are such poor substrates for ataxin-3 (Burnett and Pittman, 2005; Winborn et al., 2008). It would also not be unusual: the affinities of several isolated UBDs with mono-ubiquitin are weak, with dissociation constants >100 μM (Reyes-Turcu and Wilkinson, 2009). Yeast OTU1, for instance, shows no binding up to 2 mM ubiquitin while UCH-L3 binds ubiquitin with an affinity of 100–500 μM. As in other DUBs, the affinity of ataxin-3 will be increased by avidity thanks to the multiple binding sites, thus explaining the need of such redundancy. This third ubiquitin binding site, being rarely populated if at all when site 1 is not ubiquitinated, would become populated and active only when site 1 is occupied. Ubiquitin binding to site 1 could be completely ineffective or, perhaps more likely, less effective than the alternative site leading to the observed low enzymatic activity with small substrates.

It was previously shown that specific, engineered Josephin mutations also result in enzyme activation (Weeks et al., 2011). These mutations were generated based on the observation that the ataxin-3 like protein ATXN3L is a significantly more efficient enzyme than ataxin-3, despite the two proteins sharing 85% sequence identity. As few as three mutations, namely S12F, R59L, and T60A, are sufficient to increase the catalytic activity of ataxin-3 to levels comparable to that of ATXN3L. We can suggest an explanation for these results based on our findings: as mentioned in the original account, S12 is close to the catalytic C14 and, together with F74, may form a hydrophobic lid of the active site. It was argued that the S12F mutation could result in activation by enhancing the nucleophilicity of the active site cysteine and/or enhancing affinity for substrate (Weeks et al., 2011). In support to this hypothesis, we observe that S12 is in a conformational exchange and that ubiquitination influences its environment (i.e., different chemical shift). The other two mutations that markedly enhance ataxin-3 activity, R59L and T60A, are far from the catalytic site and in the middle of helix α3. Because these residues point far away from the interface with ubiquitin and make no direct contact with ubiquitin in the crystal structure, it was suggested that the mode of ubiquitin recognition in ATXN3L and ataxin-3 could be slightly different. Based on our data, we suggest instead that the mutations could affect the hairpin flexibility thus producing effects equivalent to those obtained by mono-ubiquitination.

In conclusion, the data presented in this work offer a new model of the mechanisms governing the catalytic activity of ataxin-3 and indicate the importance of identifying and characterizing further the multiple binding properties of this unusual DUB enzyme. Efforts along this line are currently in progress in our group.

### Conflict of interest statement

The authors declare that the research was conducted in the absence of any commercial or financial relationships that could be construed as a potential conflict of interest.

## Supplementary material

Figure S1 **Effects of the addition of free ubiquitin to JosK117-only W87R and JosK117-only I77R/Q78R, respectively**.

Figure S2 **Effects of the addition of free ubiquitin to JosK117-only W87R and JosK117-only I77R/Q78R, respectively**.

Figure S3 **Raw data for the kinetics of Ub-AMC cleavage as in Figure 8. (A)** Comparison of the cleavage rate of ubiquitin-AMC by wild-type Josephin (light blue), JosK117-only (blue), and mono-ubiquitinated JosK117-only (green). In black, a control is run without Josephin in the mixture. **(B)** Comparison of the cleavage rate of JosK117-only (blue) and mono-ubiquitinated JosK117-only (green) with that of another typical DUB, isopeptidase T (magenta). **(C,D)** Cleavage of Ub-AMC by respectively JosK117-only and mono-ubiquitinated JosK117-only in the presence of increasing concentrations of free ubiquitin. The coordinates of the model structure of mono-ubiquitinated JosK117-only can be found online at: https://bioinformatics.cineca.it/PMDB/main.php as “K117 mono-ubiquitinated Josephin domain of ataxin-3.”
