# Supplementary figures and images for: Allosteric regulation of deubiquitylase activity through ubiquitination
Source: Front Mol Biosci. 2015 Feb 5;2:2. doi: 10.3389/fmolb.2015.00002 (PMC4428445; doi:10.3389/fmolb.2015.00002)

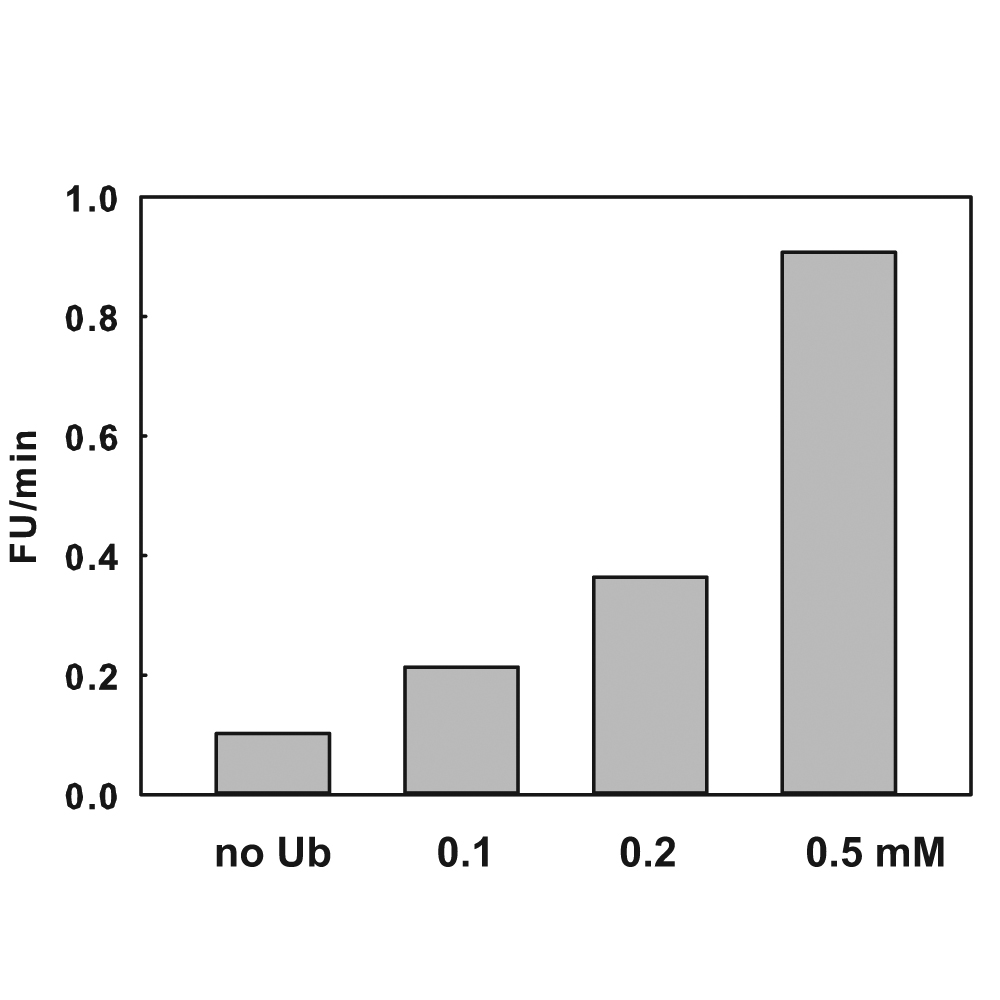

Supplement: Figure S1 — Effects of the addition of free ubiquitin to JosK117-only W87R and JosK117-only I77R/Q78R, respectively. [file FigureS1.JPEG]

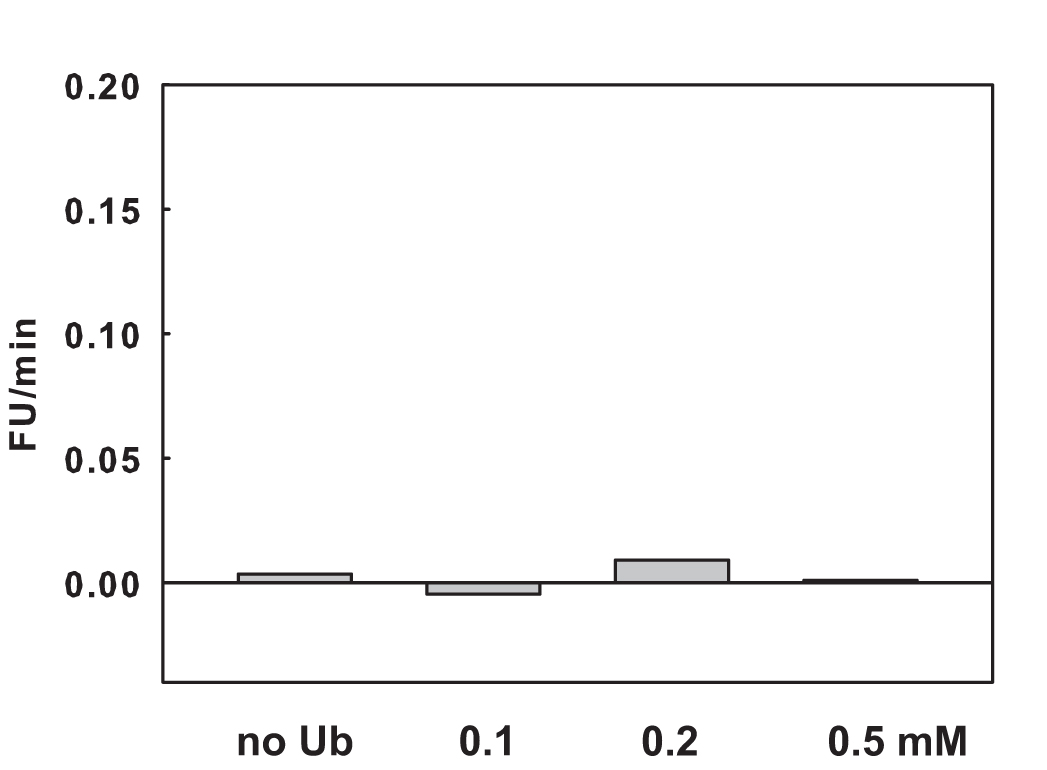

Supplement: Figure S2 — Effects of the addition of free ubiquitin to JosK117-only W87R and JosK117-only I77R/Q78R, respectively. [file FigureS2.JPEG]

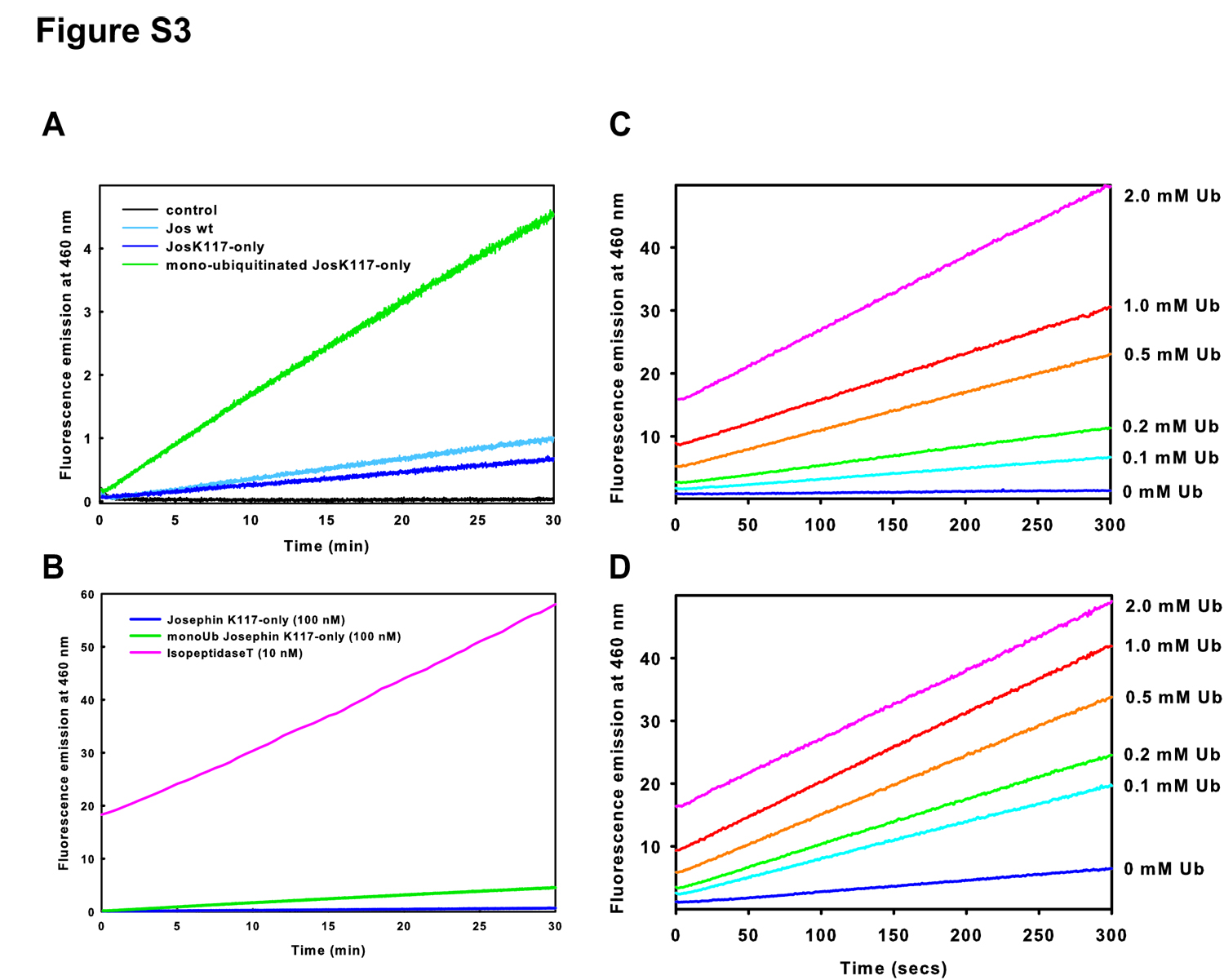

Supplement: Figure S3 — Raw data for the kinetics of Ub-AMC cleavage as in Figure 8. (A) Comparison of the cleavage rate of ubiquitin-AMC by wild-type Josephin (light blue), JosK117-only (blue), and mono-ubiquitinated JosK117-only (green). In black, a control is run without Josephin in the mixture. (B) Comparison of the cleavage rate of JosK117-only (blue) and mono-ubiquitinated JosK117-only (green) with that of another typical DUB, isopeptidase T (magenta). (C,D) Cleavage of Ub-AMC by respectively JosK117-only and mono-ubiquitinated JosK117-only in the presence of increasing concentrations of free ubiquitin. The coordinates of the model structure of mono-ubiquitinated JosK117-only can be found online at: https://bioinformatics.cineca.it/PMDB/main.php as “K117 mono-ubiquitinated Josephin domain of ataxin-3.” [file FigureS3.JPEG]
