# Supplementary material for: Allosteric regulation of deubiquitylase activity through ubiquitination
Source: Front Mol Biosci. 2015 Feb 5;2:2. doi: 10.3389/fmolb.2015.00002 (PMC4428445; doi:10.3389/fmolb.2015.00002)
Supplement: Supplementary file 4 [file Table1.PDF]

Scheme 1

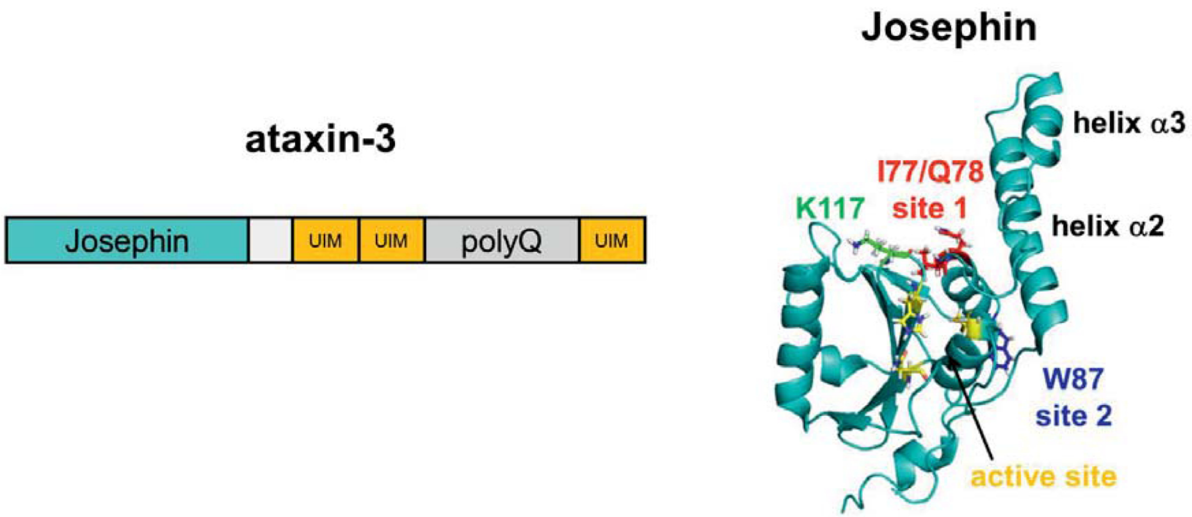

	ACTIVITY	AFTER UBIQUITINATION
Ataxin-3	K63 preference (UIMs) (Winborn et al., 2008)	increased activity (both K48 and K63 chains) (Todi et al., 2009)
Josephin	K48 preference (Todi et al., 2009)	increased activity towards K48 chains (Todi et al., 2009)
Josephin I77Q78 mutant	inactive (Nicastro et al., 2010)	poor activity (Todi et al., 2010)
Josephin W87 mutant	reduced K48 cleavage (Nicastro et al., 2010)	increased activity (Todi et al., 2010)
